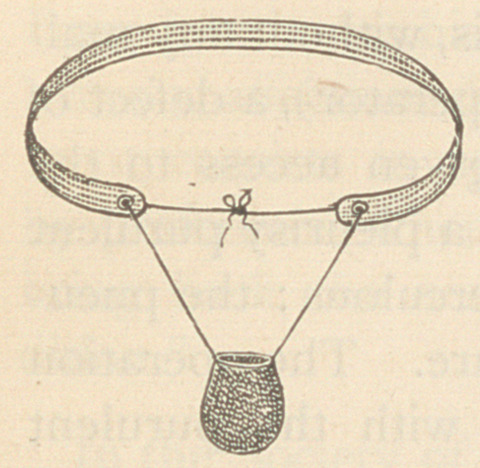# A Suspensory

**Published:** 1880-02

**Authors:** Basil M. Wilkerson

**Affiliations:** Baltimore, Md.


ARTICLE V.

### A SUSPENSORY.

BY BASIL M. WILKERSON, M. D., D. D. S., BALTIMORE, MD.

This engraving illustrates a suspensory, bag and band, invented by the writer, and which has been used by several physicians, all of whom report very favorably as to its efficiency. His object here is to give it to the profession, hoping that it may prove worthy of attention and a benefit to suffering humanity.

It consists of a bag suspended from a belt by two strings or tapes, and is made in the following manner, viz: Take a piece of muslin for the belt (four ply), about an inch wide, and of sufficient length to come within about six inches of meeting around the body, just below the crests of the ilium, on each side of the hips. Work a smooth eyelet-hole (hoop stitch) in each end, where it is best to have the goods six or eight ply to prevent wearing the strings as they slide through the eyelets for adjustment as the body takes different positions. Make a bag of knit or stocking goods, of suitable size, with slightly contracted opening or mouth, around which should be sewn a suitable binding. Sew to each side of its upper border a corset (or similar) string of sufficient length.

The suspensory is applied by placing the belt around the body below the hips, and putting the scrotum into the bag, then passing the strings through the eyelets in the belt and tying them across the abdomen to such a tension as will be comfortable to the wearer.

When the belt and bag are made the right length and size, and properly adjusted, it will be found very pleasant to a patient who has not been accustomed to wearing a suspensory.

## Figures and Tables

**Figure f1:**